# *In Vivo* Photoacoustic and Fluorescence Cystography Using Clinically Relevant Dual Modal Indocyanine Green

**DOI:** 10.3390/s141019660

**Published:** 2014-10-21

**Authors:** Sungjo Park, Jeesu Kim, Mansik Jeon, Jaewon Song, Chulhong Kim

**Affiliations:** 1 School of Electronics Engineering, College of IT Engineering, Kyungpook National University, 1370, Sankyuk-dong, Buk-gu, Daegu 702-701, Korea; E-Mails: sjpark2686@gmail.com (S.P.); jwsong@ee.knu.ac.kr (J.S.); 2 Departments of Creative IT Engineering and Electrical Engineering, Pohang University of Science and Technology, 77 Cheongam-Ro, Nam-Gu, Pohang, Gyeongbuk 790-784, Korea; E-Mails: hybridjs@postech.ac.kr (J.K.); msjeon@postech.ac.kr (M.J.)

**Keywords:** photoacoustic tomography, fluorescence image, cystography, indocyanine green

## Abstract

Conventional X-ray-based cystography uses radio-opaque materials, but this method uses harmful ionizing radiation and is not sensitive. In this study, we demonstrate nonionizing and noninvasive photoacoustic (PA) and fluorescence (FL) cystography using clinically relevant indocyanine green (ICG) *in vivo*. After transurethral injection of ICG into rats through a catheter, their bladders were photoacoustically and fluorescently visualized. A deeply positioned bladder below the skin surface (*i.e.*, ∼1.5–5 mm) was clearly visible in the PA and FL image using a laser pulse energy of less than 2 mJ/cm^2^ (1/15 of the safety limit). Then, the *in vivo* imaging results were validated through *in situ* studies. Our results suggest that dual modal cystography can provide a nonionizing and noninvasive imaging tool for bladder mapping.

## 1. Introduction

In radiology and urology, cystography is a gold standard imaging procedure to visualize urinary bladders. In conventional cystography, radio-opaque dyes (*i.e.*, X-ray contrast agents) are transuretherally injected through urinary catheters, and then fluoroscopic X-ray bladder images can be acquired [1,2]. However, this modality is ionizing and not sensitive.

Photoacoustic tomography (PAT) is a nonionizing and noninvasive hybrid imaging technique that takes advantages of the features of light and ultrasound. PAT can deliver strong optical absorption contrast and high ultrasound spatial resolution in deep tissues [3]. When a short pulsed laser (*i.e.*, typically on the order of nanosecond) illuminates biological samples, the targets absorb the light beam. Then, the absorbed optical energy is converted into thermal energy via thermoelastic expansion. Consequently, wideband ultrasonic waves (*i.e.*, referred to as photoacoustic (PA) waves) are generated. The generated PA waves travel through the tissue, and are acquired by a conventional ultrasound transducer. The PA signal amplitudes are proportional to optical energy deposition (*i.e.*, production of optical fluence and optical absorption coefficient) [4]. Therefore, PAT is very sensitive to detect the optical absorption properties of tissues. The imaging depth of PAT can reach up to a few centimeters in biological tissues and the spatial resolution can maintain ∼1/200 in all imaging depths [5]. More importantly, PAT is completely free of ionizing radiation. Therefore, PAT is a safe imaging modality for human use, a key requirement for cystography.

Fluorescence (FL) imaging is another nonionizing optical imaging modality which has been widely used in biomedical research and clinical studies [6–8]. Specially, FL optical microscopy including confocal microscopy and multi-photon microscopy is a vital tool in biomedical research [9,10]. However, the key problem of this technique is a shallow penetration depth, typically one transport mean free path (*i.e.*, ∼1 mm). As another form, planar FL imaging has been applied to clinical studies to guide intraoperative surgeries. The key success of planar FL imaging in a surgical room is that it is extremely easy to implement. However, there is a still fundamental problem that it suffers from a poor spatial resolution due to strong light scattering.

Indocyanine green (ICG) is a clinically relevant contrast agent approved by Food and Drug Administration (FDA). ICG has been widely used in many medical practices such as cardiac output monitoring, angiography in ophthalmology, and sentinel lymph node mapping in breast cancer patients [11–14]. The optical absorption and FL spectrum of ICG is in a near-infrared (NIR) region (from 600 to 900 nm) where light can penetrate deeper [15]. The FL quantum yield of ICG is ∼10% in water. This means that 90% of the excited energy can be nonradiatively released as a form of heat. Therefore, ICG can be used as a dual modal contrast agent of PAT and planar FL imaging.

In this study, for the first time to our knowledge, we demonstrate the *in vivo* dual modal PA and FL cystographic imaging capability with transurethral injection of ICG into rats. Methylene blue, gold nanocages, and single-walled carbon nanotubes have been used as PA contrast agents for this purpose [16–18]. However, the use of ICG is the most relevant to the clinical translation. Rats' bladders filled with ICG have been photoacoustically and fluorescently imaged *in vivo*, and then the imaging results have been validated through *in situ* studies. Therefore, the dual modal PA and FL imaging can potentially be a nonionizing alternative for bladder imaging.

## 2. Methods

### 2.1. In Vivo Photoacoustic and Fluorescence Cystography Experiment Setup

**Figure 1.**
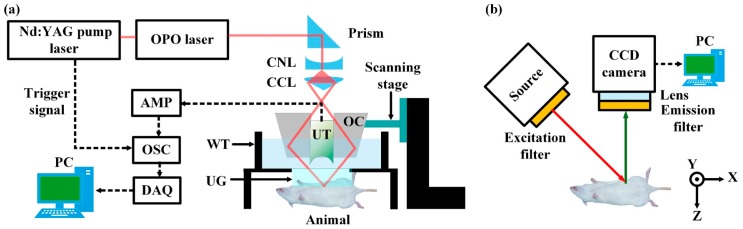
Experimental setups of a dual modal photoacoustic (**a**) and fluorescence (**b**) imaging system. AMP, Amplifier; OSC, oscilloscope; OPO, optical parametric oscillator; CNL, conical lens; CCL, concave lens; OC, optical condenser; UT, ultrasound transducer; WT, water tank; and UG, ultrasound gel.

Figure 1a,b shows the schemes of two independent PA and FL imaging systems, respectively. Tunable pulsed light with a pulse width of 5 nanoseconds was generated by an optical parametric oscillator system (Surelite OPO PLUS, Continuum, San Jose, CA, USA; wavelength tuning range, 680 ∼ 2500 nm) pumped by a Q-switched Nd-YAG laser (SLIII-10; Continuum; 532 nm). The laser repetition rate was 10 Hz. The optical wavelengths of 800 and 950 nm were used for spectroscopic bladder identification. The laser beam was delivered to a spherical conical lens through several right angle prisms (PS908, Thorlabs, Newton, NJ, USA). After passing through the spherical conical lens, the ring-shaped laser beam pattern was formed. The diverged donut-shaped beam pattern was refocused by a hand-made optical condenser, and then was incident onto rats positioned under a water tank. The bottom of the water tank was wrapped with a thin membrane which is optically and acoustically transparent. When the pulsed laser beam illuminated the rats, PA waves were generated.

The induced PA waves propagated in water, and then were captured by a single-element spherically focused ultrasound transducer (V308; Olympus NDT, Waltham, MA, USA; a center frequency of 5 MHz). The ultrasound transducer was located in the middle of the hand-made optical condenser and the ultrasound focus was co-axially aligned with the optical focus to improve signal-to-noise ratios (SNRs). The laser pulse energies used were 1.9 and 1.5 mJ/cm^2^ at 800 and 950 nm, respectively. The laser safety limits at 800 and 950 nm were 31.7 and 63.2 mJ/cm^2^, respectively. The spatial resolutions were 144 and 590 μh in the transverse and axial directions, respectively. The acquired PA signals were amplified by a pulser/receiver (5072PR; Olympus, Tokyo, Japan) and recorded by a mixed signal oscilloscope (MSO 5204; Tektronix, Beaverton, OR, USA) with a sampling rate of 50 × 10^6^ samples/second. By moving the optical condenser with a linear stage along the *x* axis, we were able to acquire cross-sectional B-mode PA images in an image plane of *x* and *z* axes. Additional raster scanning along the *y* axis enabled us to obtain volumetric PA images. The imaging acquisition time is ∼35 min for one volumetric PA image with a field of view of 4.6 × 4.6 cm^2^ along the *x* and *y* axes, respectively. No signals were averaged. The PA data was processed through maximum amplitude projection (MAP) in the corresponding *x*-*y* plane.

Figure 1b shows a FL imaging system. We used a high-power light-emitting diode (LED; M780L3; Thorlabs) with a center wavelength of 780 nm as an excitation light source. After passing through an excitation filter (ET775/50x; 28 nm bandwidth; CHROMA, Windham, VT, USA), the excitation light illuminated the sample. Emitted FL light first past through an emission filter emission filter (ET845/55m; CHROMA), and then was detected by a NIR CCD camera.

### 2.2. Animal Handling

All animal experimental procedures were performed in accordance with protocols approved by an institutional animal care and use committee (IACUC). Healthy Sprague-Dawley female rats weighing approximately ∼220 g were used in all *in vivo* imaging experiments. We anesthetized the rats using vaporized isoflurane (1 L/min of oxygen and 0.75% isoflurane). After shaving abdominal hairs, the rats were located atop a hand-made animal holder. A 22-gage lubricant-coated catheter was inserted into a bladder through urethral opening, and urine in the bladder was voided via the catheter. Before injection of ICG, we acquired control PA and FL images. Then, ICG with a concentration of 1 mM and a dose of 0.4 mL was transurethrally injected, and a series of PA and FL images were obtained. After all *in vivo* imaging experiments, the rats were sacrificed with an overdose of pentobarbital, and the *in vivo* imaging results were validated through *in situ* studies.

## 3. Results

**Figure 2.**
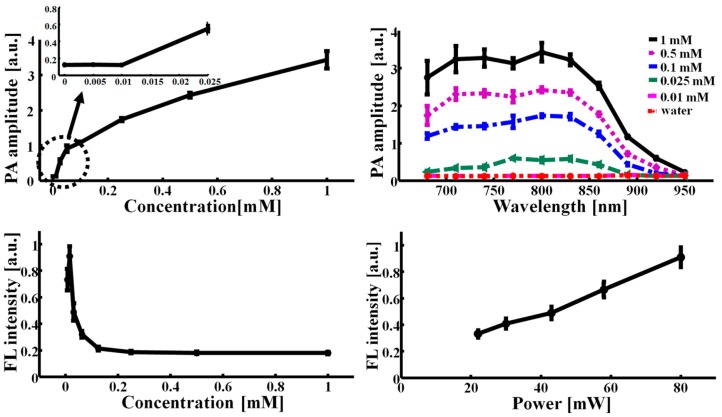
Photoacoustic (PA) and fluorescence (FL) properties of indocyanine green (ICG). (**a**) PA amplitudes *vs.* ICG concentration; (**b**) PA spectra of ICG by varying concentration; (**c**) FL intensities *vs.* ICG concentration; (**d**) FL intensities *vs.* LED output power.

To investigate the optical properties of ICG, we first measured the PA sensitivity and spectrum *in vitro*. The concentration of ICG was varied from 0 to 1 mM (*i.e.*, 0, 0.01, 0.025, 0.1, 0.5, and 1 mM) and the optical wavelength was tuned from 680 to 950 nm. As the ICG concentration increased, the PA signal linearly increased at the beginning phase, and then the profile became slightly nonlinear (Figure 2a). We were able to detect the PA signal at the concentration of 25 μM with a SNR of 7.5 ± 0.1. The noise equivalent sensitivity is approximately 11 μM, and well agrees with the previously reported result [14]. In the PA spectrum, the peak PA signal was generated at 800 nm, which is well within the NIR spectral region for deep penetration (Figure 2b) [19]. Then, the PA signal decreased as the wavelength was swept to longer wavelengths, and then eventually the PA signal was not detectable at 950 nm despite the use of the highest concentration (*i.e.*, 1 mM). Therefore, the ICG concentration of 1 mM was used for *in vivo* PA imaging, and the two wavelengths (*i.e.*, 800 *vs.* 950 nm) were switched for spectroscopic PA bladder identification. Furthermore, we measured the FL intensities of ICG by varying the concentration from 7 μM to 1 mM (Figure 2c). The FL intensity initially increased, peaked at a concentration of 15 μM, and then quickly decreased. Beyond the concentration of 0.25 mM, the FL intensity was not detectable. This result implies that ICG is highly aggregated at higher concentration and consequently the FL quantum yield is reduced. Thus, the nonradiative energy decay becomes dominant as an energy relaxation mechanism. In other words, the heat conversion efficiency of the ICG molecules can be significantly enhanced. Further, we confirmed that the FL intensities increased as the LED power mounted (Figure 2d).

**Figure 3.**
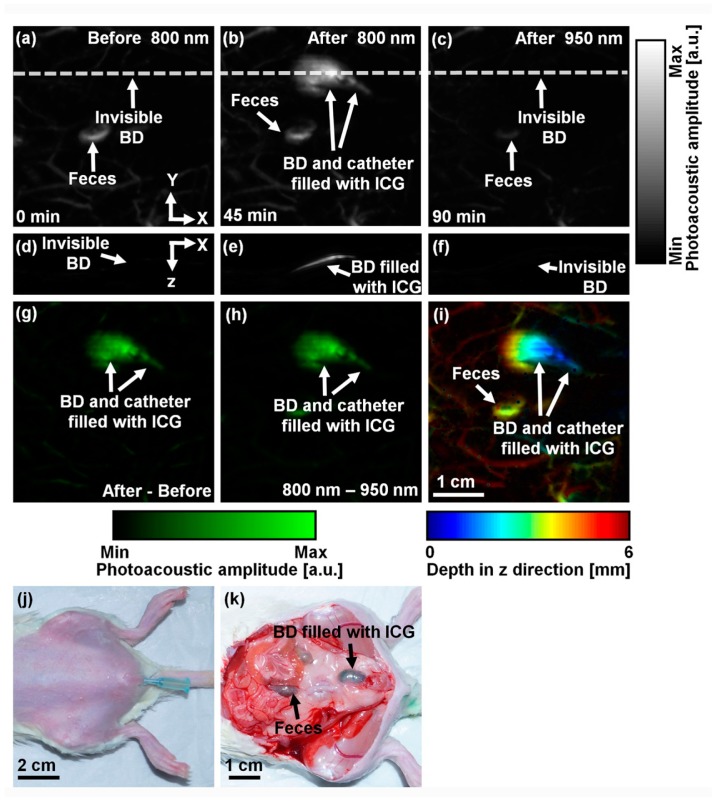
*In vivo* nonionizing and noninvasive photoacoustic (PA) cystography using indocyanine green (ICG). (**a**) Control PA MAP image of a rat's abdomen at 800 nm acquired before ICG injection. Only surround blood vessels are visible; (**b**) PA MAP image at 800 nm after ICG injection displaying the bladder (BD) filled with ICG, catheter, and blood vessels; (**c**) PA MAP image at 950 nm after ICG injection. The bladder disappeared due to the spectral response of ICG; (**d**–**f**) Depth-sensitive B-mode PA images of (a–c) cut along the dotted lines; (**g**) PA differential MAP image between (a) and (b); (**h**) PA differential MAP image between (b) and (c); (**i**) Depth-sensitive pseudo-colored PA MAP image of (b); (**j**) Photograph taken before PA imaging; (**k**) Photograph taken after PA imaging and skin removal.

To explore *in vivo* PA cystography with ICG, we acquired a control PA image at a wavelength of 800 nm before ICG injection. The photograph of a transuretheral catheterized rat is shown in Figure 3j. Figure 3a shows the control PA MAP image of the rat's abdomen. Only blood vessels and feces are clearly visible, but the bladder is invisible because of the optical transparency of urine. The PA contrast within the region of the bladder is 0.2 ± 0.1 (*i.e.*, PA contrast compared to the background = (PA_bladder_ − PA_background_)/ PA_background_). The averaged PA contrast of the surrounding blood vessels is 2.0 ± 0.5. After transurethral injection of ICG with a concentration of 1 mM and a dose of 0.4 mL, the bladder and catheter tip filled with ICG are obviously delineated with a PA contrast of 21.0 ± 4.5 (Figure 3b). The PA contrast of the bladder compared to the surrounding blood vessels (*i.e.*, PA_bladder_ − PA_blood vessels_)/ PA_blood vessels_) is 4.1 ± 0.7. This result implies that the optical absorption of extrinsic ICG is dominant compared to that of the intrinsic hemoglobin, an important fact for future clinical applications. To confirm the accumulation of ICG in the bladder, we switched the optical wavelength to 950 nm. Because ICG does not absorb the light at this wavelength, the bladder and catheter disappeared in the PA image (Figure 3c). Figure 3d–f shows the corresponding depth-resolvable B-mode PA images of Figure 3a–c, respectively, cut along the dotted lines. The bladder is only visualized in the B-mode PA image acquired at 800 nm after ICG injection (Figure 3e). To further enhance the PA image contrast, we processed the PA images as follows: (1) the difference between the two PA images acquired at pre- and post-injection (*i.e.*, Figure 3g, the subtraction of Figure 3a from Figure 3b) and (2) the difference between the two PA images obtained at 800 and 950 nm (*i.e.*, Figure 3h, the subtraction of Figure 3c from Figure 3b). The PA image contrasts in Figure 3g,h are 64.0 ± 31.4 and 151.8 ± 77.1, respectively, and the contrasts are 3.1 and 7.4 times higher than that calculated from Figure 3b, respectively. We mapped the depth information of the PA image (Figure 3b) using pseudo colors (Figure 3i). The bladder is located approximately ∼1.5–5 mm from the skin surface. After all *in vivo* PA imaging experiments, the rat was sacrificed and the bladder was exposed for validation. As shown in Figure 3k, the greenish bladder confirmed the ICG accumulation.

**Figure 4.**
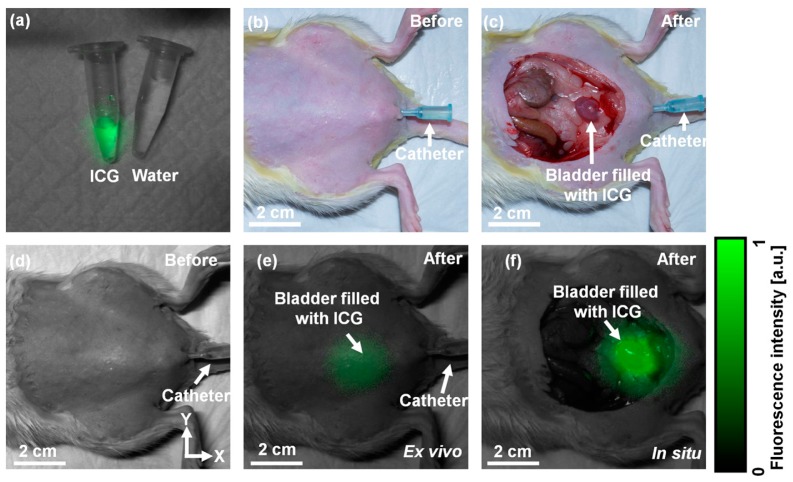
*In vivo* nonionizing and noninvasive fluorescence (FL) cystography using indocyanine green (ICG). (**a**) Combined FL and white-light image of ICG (15 μM) and water in plastic tubes; (**b**) Photograph taken before FL imaging and ICG injection; (**c**) Photograph taken after FL imaging and skin removal; (**d**) Control overlaid FL and white-light image of the rat's abdomen before ICG injection; (**e**) *In vivo* overlaid FL and white-light image after ICG injection; (**f**) *In situ* overlaid FL and white-light image after ICG injection and skin removal. BD, bladder.

By using the planar FL imaging system, the accumulation of ICG was monitored. Figure 4a shows the overlaid FL and white-light images of ICG and water in plastic tubes. The concentration of ICG was 15 μM. Figure 4b is the photograph of the rat after hair removal and catheterization. Figure 4d and e is the combined FL and white-light images of the rat's abdomen before and after ICG injection (*i.e.*, 15 μM and 400 mL), respectively. The bladder filled with ICG was fluorescently visualized after ICG injection (Figure 4e), while no FL light was captured in the control image (Figure 4d). The normalized FL signal was 0.28 ± 0.02 (a.u.). Unlike the PA image, the FL image suffers from the poor spatial resolution due to strong light scattering in the animal. Then, the rat was sacrificed and the abdominal skin was removed. The accumulation of ICG was validated through *in situ* FL imaging as shown in Figure 4f. Certainly, the normalized FL light intensity was greatly enhanced to 0.99 ± 0.03 (a.u.) because the bladder was exposed (Figure 4c).

## 4. Conclusions

In conclusion, we have successfully demonstrated nonionizing and noninvasive dual modal photoacoustic and fluorescence cystography employing clinically relevant ICG in small animals *in vivo*. By injecting ICG transurethrally, the rats' bladders were photoacoustically and fluorescently imaged. Then, we further confirmed our *in vivo* imaging results through the *in situ* studies. The clinical PA imaging probe adapted with clinical ultrasound imaging systems has recently been developed, and a few prototype system has been used in clinical studies [20]. Thus, we believe that the clinical translation of our approach is highly possible.
